# Extent and direction of introgressive hybridization of mule and white‐tailed deer in western Canada

**DOI:** 10.1111/eva.13250

**Published:** 2021-06-01

**Authors:** Ty Russell, Catherine Cullingham, Mark Ball, Margo Pybus, David Coltman

**Affiliations:** ^1^ Department of Biological Sciences University of Alberta Edmonton Canada; ^2^ Department of Biology Carleton University Ottawa Canada; ^3^ Alberta Fish and Wildlife Edmonton Canada; ^4^ Present address: LGL Limited Environmental Research Associates Sidney Canada

**Keywords:** chronic wasting disease, deer, hybridization, introgression, single nucleotide polymorphism, ungulate

## Abstract

Hybridization of mule deer (*Odocoileus hemionus*) and white‐tailed deer (*O*. *virginianus*) appears to be a semi‐regular occurrence in western North America. Previous studies confirmed the presence of hybrids in a variety of sympatric habitats, but their developing molecular resources limited identification to the earliest, most admixed generations. For this reason, estimates of hybrid production in wild populations often rely on anecdotal reports. As well, white‐tailed deer populations’ continued encroachment into historically mule deer‐occupied habitats due to changes in land use, habitat homogenization, and a warming climate may increase opportunities for interspecific encounters. We sought to quantify the prevalence and extent of hybrid deer in the prairies of western Canada using a SNP assay with enhanced discriminating power. By updating the available molecular resources, we sought to identify and characterize previously cryptic introgression. We also investigated the influence of various parameters on hybridity by way of logistic regression. We observed overall hybridization rates of ~1.0%, slightly lower than that reported by previous studies, and found white‐tailed‐like hybrids to be more common than their mule deer‐like counterparts. Here, we build upon past studies of hybridization in North American deer by increasing hybrid detection power, expanding sample sizes, demonstrating a new molecular resource applicable to future research and observing asymmetrical directionality of introgression.

## 1 INTRODUCTION

Initial steps towards divergence and speciation involve the accumulation of reproductive barriers (Kirkpatrick & Ravigné, 2002; Orr & Turelli, 2001). Even in the presence of gene flow, prezygotic barriers between taxa can develop from extrinsic factors (niche divergence, sexual selection, behavioural isolation, etc.) or strong genetic drift to eventually establish postzygotic, ecologically independent isolation (Abbott et al., 2013; Hewitt, 2001; Nosil, 2008; Wang & Bradburd, 2014). Both the building up and tearing down of these isolating mechanisms have been topics of interest among historical and contemporary evolutionary biologists. The blurring of genetic boundaries between divergent taxa, especially at the species level, is called hybridization and can give rise to a wide variety of outcomes. These can include progressive evolutionary events, such as novelty, disruptive selection, and speciation (Dowling & Secor, 1997; Lamer et al., 2015; Seehausen, 2004), as well as consequences that interfere with management and conservation efforts by compromising co‐adapted gene complexes, morphological discernment, local adaptation, and the genetic integrity of unique phylogenetic lineages (Edmands et al., 2009; Leary et al., 1996; Martinsen et al., 2001; Rhymer & Simberloff, 1996).

Historically, one of the most essential requisite components in the study of hybrid populations has also been its most problematic roadblock: the ability to reliably identify hybridized individuals. Characterizing hybrid offspring using morphological markers alone is not ideal because hybrid physiologies may not always be intermediate composites of parental forms (Leary et al., 1996; Rhymer & Simberloff, 1996). In particular, gene expression patterns often exhibit increased variation in systems with extensive introgression (Grant & Grant, 1994; Knief et al., 2019; Lu et al., 2018). When morphological data are available for the study of hybridized populations, it is best used in conjunction with genetic data. Furthermore, since introgression of foreign alleles may be distributed unevenly throughout the genome, loci should be numerous with highly differentiated allele frequencies between species (Muirhead & Presgraves, 2016; Randi et al., 2014). Although short tandem repeat microsatellite markers are often more informative on a per‐locus basis (owing to their allelic diversity; Fernández et al., 2013), single nucleotide polymorphisms (SNPs) are quickly becoming the marker of choice for interrogating admixture. SNPs are well‐suited to hybrid studies in two important ways: high abundance in the genome promotes the discovery of loci with alleles that are species‐specific, or at least highly differentiated between species (Cullingham et al., 2013; Lamer et al., 2014; Stephens et al., 2009; Twyford & Ennos, 2011; Wiley et al., 2009), plus their biallelic nature simplifies the differentiation of two species (i.e. by recognizing that loci with fixed differences possess a species A allele and a species B allele).

Mule deer (*Odocoileus hemionus*; MD) and white‐tailed deer (*O*. *virginianus*; WT), like most hybridizing species, are closely related and sympatric over large parts of their ranges (Abbott et al., 2013; Bradley et al., 2003; Cronin, 1991; Gourbière & Mallet, 2010; Hornbeck & Mahoney, 2000; Price & Bouvier, 2002; Stelfox & Adamczewski, 1993). Although both are designated as species of least concern as of 2021 (iucnredlist.org), they face impending challenges from chronic wasting disease (CWD) and anthropogenic habitat disturbance. CWD is a transmissible spongiform encephalopathy akin to mad cow in cattle (bovine spongiform encephalopathy) and Creutzfeldt–Jakob disease in humans (Cullingham et al., 2011; Miller et al., 2004, 2012; Williams, 2005; Williams et al., 2002). While social structure and behaviour have proven to be important factors impacting the horizontal spread of CWD within species (Cullingham et al., 2010, 2011), the consequences of interspecific encounters—including hybridization—have not been well studied. As well, recent and ongoing anthropogenic changes to cervid habitat have influenced home range shifts (Fisher et al., 2020). As home ranges change over time so do the ranges of species overlap, which may promote interspecific contact and opportunities for hybridization. Specifically, large‐scale industrial development causes diverse landscapes to homogenize, promoting early seral vegetation and reducing sloped and forested areas, largely to the benefit of WT and the detriment of MD and caribou (*Rangifer tarandus*) (DeCesare et al., 2010; Fisher & Burton, 2020). The proliferation and encroachment of WT into habitat formerly dominated by MD may affect species interactions on a systematic scale. For example, the bidirectional nature of MD x WT hybridization is well‐documented, but shifting local species compositions may affect the dynamics of contemporary introgression (Ballinger et al., 1992; Bradley et al., 2003; Cronin, 1991).

Molecular markers previously developed for the study of MD × WT hybrids include serum albumin electrophoresis (McClymont et al., 1982), a ribosomal 28S DNA marker (Bradley et al., 2003), and mitochondrial endonuclease recognition site mapping (Carr et al., 1986). These methods facilitated a number of discoveries: past admixture with black‐tailed deer (*O*.* h*. *columbianus*) (Carr & Hughes, 1993; Latch et al., 2011), adherence to Haldane's rule (Hornbeck & Mahoney, 2000; Wishart et al., 1988), and evidence of bidirectional introgression (Ballinger et al., 1992; Bradley et al., 2003; Cronin, 1991). Russell et al. (2019) increased the power of hybrid detection by incorporating 40 species‐specific SNPs into an assay that provides a highly diagnostic measure of hybridity. By building upon past molecular resources, we hope to unlock previously inaccessible levels of introgression. Resolution of backcross generations is a helpful advancement in this system because Wishart et al. (1988) found that female F1 hybrids often remain fertile, while male fertility is only likely to return after several backcrosses. Without a considerably powerful method of detection, post‐F1 hybrids often remain hidden because the proportion of their genome inherited from one species is, on average, halved at each backcross (Boecklen & Howard, 1997). This causes heterospecific alleles to decrease logistically with subsequent backcross generations, resulting in progressively cryptic hybrid landscapes.

Our goals for this study were to comprehensively survey the state of hybrid deer in Alberta, identify the directional trends of introgression and investigate which parameters affect the frequency and/or direction of hybridization. To realize these objectives, we used a panel of 40 diagnostic SNPs (Russell et al., 2019) to genotype individuals selected by a sampling method that accounted for putative species, CWD status, sex, and geographic locality. We then evaluated hybridity using *NewHybrids* version 1.1 beta (Anderson & Thompson, 2002) and *STRUCTURE* version 2.3.4 (Pritchard et al., 2000) before running a logistic regression model to determine which conditions influence hybridization dynamics. We hope our results will provide insights for researchers seeking to optimize management policies and forecast systematic trends of hybridizing ungulate populations.

## 2 METHODS

### 2.1 Sampling

All deer samples genotyped by SNP assay were collected from hunter‐harvested animals that were submitted to the mandatory CWD surveillance programme in Alberta, Canada, which monitors the spread and prevalence of the disease. Tissue samples were collected primarily using ear punches obtained through tagging or from skeletal muscle taken from deceased individuals and stored at −20°C. Because the number of deer tested for CWD each year exceeds the scope of this study, we sampled individuals using four different grouping strategies, which we will refer to using the following terms: demographic‐matching, disease‐matching, foothills, and ambiguous species. Since the majority of our samples were submitted by hunters, we took these measures to minimize ascertainment bias caused by uneven distribution of sampling location, sex, species, and CWD status. The initial species identification during collection was provided by the hunter contributing the sample (visual identification; harvest licences are species‐specific) and recorded by Alberta Fish and Wildlife staff.

**FIGURE 1:**
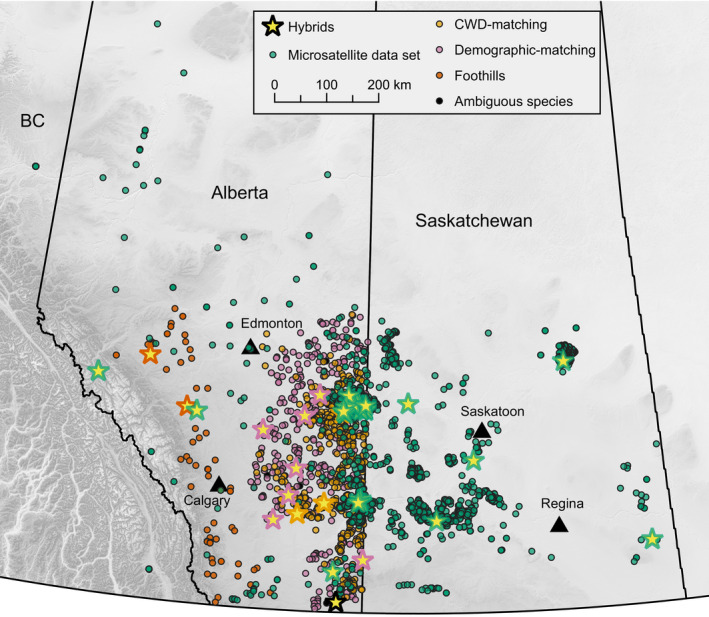
Collection localities of all samples. All individuals genotyped at SNP loci were from Alberta, some in the microsatellite data set were from BC and Saskatchewan. Outline colour of the star shape indicates the group from which a hybrid was sampled. See Table 1 for a distribution of sample group membership

The purpose of the demographic‐matching group was to randomly sample deer from a large pool while ensuring coverage of the study area and balancing sex and species. For this approach, the study region (~230,000 km^2^ of southern Alberta; Figure 1) was divided into a grid of 9 × 17 cells of equal size, 101 of which contained at least one sample of each sex of each species (two concessions were made due to lack of female WT; Table 1). From those 101 cells we randomly selected one of each of the following: male MD, female MD, male WT, and female WT. Thus each sample was one of four different demographic categories from approximately the same geographic location (*n* = 404).

**TABLE 1:** Breakdown of sample group membership. Species and sex of individuals composing subsample groups

Sampling group	MD males	MD females	WT males	WT females	Males	Females	Sex NA	MD	WT	CWD+	CWD‐	Group totals
**Demographic‐matching**	102	99	101	102	203	201	–	201	203	0	404	404
**Disease‐matching**	326	117	39	14	365	131	–	444	53	249	248	497
**Foothills**	11	7	23	29	34	36	–	18	52	0	70	70
**Ambiguous species**	–	–	–	–	7	8	–	–	–	0	16	16
Microsatellite group	1202	1486	841	1132	2043	2618	335	2914	2082	–	–	4996
Column total	1641	1709	1004	1277	2652	2994	335	3577	2390	249	738	5983

Note

The sexes of one CWD+ mule deer and one ambiguous species were unknown and are reflected in the totals. Groups in bold were genotyped at species‐diagnostic SNPs.

The disease‐matching group was designed with a similar pairing‐up procedure. 250 deer tissue samples on hand had previously tested positive for CWD. We paired each of these individuals with one same‐sex conspecific CWD‐negative from the same wildlife management unit (randomly, when more than one was available). This resulted in an uninfected neighbour whose sex and species were matched to each CWD‐positive deer (*n* = 500). By controlling for sex, species, and geography of our pool of samples, we could more objectively test whether disease status and hybridization rate held any association within this group via logistic regression and Fisher's exact test. Note that sex ratio is not balanced evenly in this group but reflects the sex ratio of the CWD+ individuals.

The foothills group was not filtered and consisted of samples (*n* = 70) from western Alberta, near the Rocky Mountains (Figure 1). This region represents the convergence of alpine and prairie habitats. We theorized that interspecific mating events may be increased in areas where the flat, gentle terrain preferred by WT meets the rugged, irregular terrain frequented by MD (Lingle, 2002).

The ambiguous species group consisted of those individuals whose species could not be confidently discerned by the hunter submitting the sample (*n* = 16). We included these in our analyses because ambiguous or intermediate morphological markers are common in animals of hybrid ancestry (Bachanek & Postawa, 2010; Mavárez et al., 2006). All random sampling was performed using R statistical software version 3.6.3 and the base functions therein (R core team, 2020). An additional group of *a priori* hybrids (*n* = 72) from a long‐term captive breeding study (Wishart et al., 1988) were also genotyped as a reference and analysed separately from the above empirical samples. Since the pedigree of these individuals is known, the results of their hybrid assignments will further validate the assay as positive controls.

All samples described above were genotyped using a 40‐loci species‐discriminating SNP assay (Russell et al., 2019). Additionally, we included data from 4,996 samples genotyped at 10 microsatellite loci generated by Cullingham et al. (2010, 2011). Although it has less diagnostic power than the SNP assay, the volume of this data set will serve as quantitative support by supplementing the sample size. For sampling, extraction and genotyping procedures, see Cullingham et al. (2010, 2011).

### 2.2 Extraction and genotyping

All DNA was extracted using a Qiagen 96 DNeasy Blood and Tissue Kit following the manufacturer's instructions (Qiagen, Mississauga, Ontario, Canada) and eluted into 150 µL of elution buffer. Reactions were performed in 96‐well plates. DNA was tested for quantity using a NanoDrop 2000 Spectrophotometer. Further DNA quantification using a QuantiFluor assay and Illumina SNP genotyping procedures was carried out by NEOGEN Genomics (NEOGEN Genomics, Lincoln, Nebraska) (Gabriel et al., 2009).

Development and validation of the SNP assay are detailed more extensively elsewhere (Russell et al., 2019) but will be briefly summarized here. A pool of various deer species, including 17 WT and 8 MD from western Canada, were genotyped using the high‐throughput 50k Cervus SNP50 (Brauning et al., 2015) Bead Chip assay (Illumina, San Diego) as part of an effort to advance the genomic resources available for the New Zealand deer farming industry (Rowe et al., 2015). In this data set, 129 loci had alternatively fixed alleles between species (i.e. all WT homozygous for allele A and all MD homozygous for allele B). These were pared down to 40 loci to fit our financial and research scopes. The assay was validated by genotyping 30 more deer: 10 MD and 10 WT from allopatric regions of Canada and the United States (see below) and 10 hybrids bred in captivity with known pedigrees (Wishart et al., 1988). Genotypes are publicly available as a supplementary table in Russell et al. (2019). Thus, the assay consists of 40 SNP loci approaching fixation in 27 WT and 18 MD from sympatric and allopatric populations and which also behaved predictably in 10 *a priori* hybrids from within the study area. Importantly, Boecklen and Howard (1997) predicted the theoretical frequency with which heterozygote genotypes appear in hybrid backcrosses, assuming fixed alleles. Using 40 perfectly discriminating SNP loci, the probability that a 3rd‐generation backcross individual will have a parental genotype (i.e. all homozygous) is 0.005; the chances of confusing a 4th backcross with a parental increase to 0.076 (taken from Eq. 3 using *n* = 40 loci, *z* = 0 heterozygote loci and *i* = 3 and 4 backcross generations, respectively).

### 2.3 Admixture analysis

Russell et al. (2019) projected the SNP assay to reliably identify third backcross generation hybrids. Building on this, we generated and analysed simulated data sets that would support our research objectives twofold: by determining which computational methods of hybrid assignment would perform well with our data sets and by calibrating the stringency of hybrid assignments into a range of realistic estimates. We recognize that in silico evaluations do not perfectly simulate in situ populations because MD and WT have been sympatric over much of their range in Alberta—and have likely shared at least some gene flow—for many generations. First, 10 MD and 10 WT from allopatric populations outside the study area were genotyped as parental species references with no interspecific gene flow (we will use the term ‘parental’ to refer to deer with no detectable admixture). This initial generation could then be used to simulate populations of varying hybridity. While 10 individuals are unlikely to capture the extent of genetic variation in an entire species, we will consider them (along with the initial 25 deer from Rowe et al. 2015) as a hypothetical parental population for comparison purposes against which we can calibrate Q‐score thresholds (below). We used the *hybridize* function from the R package *adegenet* (Jombart, 2008; Jombart & Ahmed, 2011) to create hybrid populations with three and four backcross generations. *Hybridize* samples gametes with replacement following a multinomial distribution from the given population's allele frequencies. Populations were composed of 100 individuals from each hybrid generation: parental WT, parental MD, F1, F2, first, second, third, and fourth backcrosses of each species. We then repeated this procedure and combined replicates to simplify results, such that the population with three backcrosses had *n* = 2,000 individuals and that with a fourth had *n* = 2,400. Although arbitrary, we chose these population sizes because the next step in the pipeline—Bayesian admixture analysis—is somewhat computationally intensive. This R script and the requisite genotypes of the 20 allopatric samples are available publicly on Dataverse (https://doi.org/10.7939/DVN/FSRWR4).

The simulated data sets were used as the input for a range of hybrid detection programmes: *NewHybrids* version 1.1 (Anderson & Thompson, 2002), *Snapclust* (Beugin et al., 2018), *STRUCTURE* version 2.3.4 (Falush et al., 2003; Pritchard et al., 2000; Stephens et al., 2001), and hybrid index as calculated by the R package *Introgress* (using allopatric individuals as a reference; Gompert & Buerkle, 2010). At three backcross generations, *NewHybrids* results achieved the lowest rates of false negatives (hybrids mistaken as parental) and false positives (parental mistaken as hybrids), so we chose it as our default programme. *NewHybrids* uses Bayesian model‐based clustering and a Markov Chain Monte Carlo (MCMC) algorithm to compute the posterior probabilities of assignment of individuals to specific hybrid classes (Anderson & Thompson, 2002). The classes are specified by the user as ‘genotype frequency classes’ and will vary with detection power of loci. 10 genotype frequency classes were set for the SNP data, representing the 10 generations of simulated genotypes: MD, WT, F1, F2, and three backcrosses in both directions. Similar simulations were used to evaluate the efficacy of hybrid assignment by the microsatellite suite. Because this data set offered less discriminating power, its assignments were set to 6 genotype frequency classes: MD, WT, F1, F2, and one backcross generation. *NewHybrids*, this time using 6 genotype frequency classes, again minimized error rates and was used going forward. All *NewHybrids* runs used 100,000 burn‐in reps followed by 900,000 reps for data collection.

**FIGURE 2:**
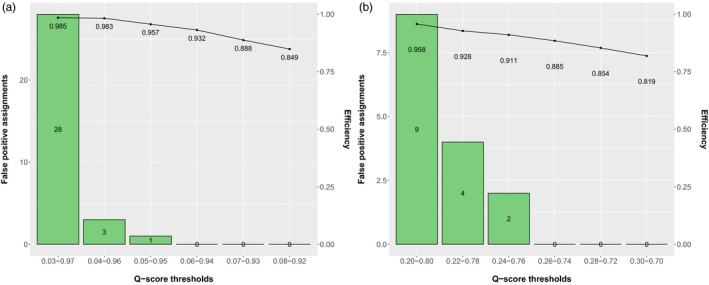
*STRUCTURE* analysis results, in terms of false‐positive assignments (parental mistaken as hybrid; represented by bars) and assignment efficiency (hybrids correctly assigned / total hybrids; represented by points), in two different data sets. Each simulated population consisted of *n* = 200 individuals from each of the following hybrid generations: parental MD, parental WT, F1, F2, and either 2 or 4 backcrosses of each species. Thresholds were imposed on the Q‐score credibility interval as a means of identifying hybrids. The most relaxed thresholds that did not commit a false positive were used, such that further widening caused false‐positive assignments and narrowing reduced efficiency. (a) In a population with 4 backcross generations, the SNP data set was optimized at thresholds 0.06 < Q < 0.94. (b) In a population with 2 backcross generations, the microsatellite suite met these criteria with thresholds of 0.26 < Q < 0.74

On the population with four backcrosses, which requires 12 genotype frequency classes, *NewHybrids* introduced occasional false positives. *STRUCTURE* performed better at this level of introgression (Figure 2). *STRUCTURE*, like *NewHybrids*, uses a Bayesian MCMC algorithm to assign individuals to clusters. In the context of hybrid studies, the two clusters correspond to the two parental species. The Q‐score metric, along with its 90% credibility interval (CI), represents the genetic contribution of each cluster as a single statistic such that 0 indicates parental WT and 1 indicates parental MD. We therefore chose to use both methods, complementing the more conservative, discrete assignments of *NewHybrids* with the more sensitive, continuous measure of the *STRUCTURE* Q‐score. All *STRUCTURE* runs used the allopatric individuals as POPDATA in the admixture model (INFERALPHA =1) with correlated allele frequencies (LAMBDA =1) and consisted of 100,000 burn‐in reps and 900,000 MCMC reps.

To implement the Q‐score as criteria for hybrid identification, we used the simulated populations to determine appropriate Q‐score cut‐offs. This way, the empirical results are compared to an idealized data set (i.e. without genetic drift, incomplete lineage sorting, rare alleles) to determine a maximum estimate of hybridity. This estimate, along with the estimate by *NewHybrids* set to 10 genotype frequency classes (6 in the microsatellite genotypes), will provide a more realistic range of hybridization rates than either programme on its own. To define thresholds on the Q‐score CI values, we identified the most relaxed range that did not produce any false‐positive assignments: 0.06–0.94 in the SNP genotypes and 0.26–0.74 for the microsatellite genotypes (Figure 2). Individuals from the empirical data set whose Q‐score CI overlapped these ranges were classified as hybrids (as in Senn et al. 2019). Summary statistics for microsatellite and SNP loci were calculated in the R package *Hierfstat* (Goudet, 2005) (Tables S1 and S2, respectively).

### 2.4 Modelling introgression direction

To identify factors that may influence direction or extent of hybridization, we ran a logistic regression model with hybrid status as the dependent variable. Hybrid status was coded as binary such that individuals within Q‐score thresholds were 1 and parental species were 0. We redefined the species variable using the results of our own admixture analysis so that those deer with Q‐scores <0.5 were WT‐like and those >0.5 were MD‐like. Using genetic evidence rather than hunters’ observations to define species allowed us to include the ambiguous species sampling group in these analyses and circumvent any identification errors of deer with intermediate morphologies. We first investigated which variables held significant associations with hybrid status by including species, sex, and CWD status as independent predictors. This model included all individuals from both SNP and microsatellite data sets. The response and all predictor variables were binary. We used a two‐sided Fisher's test to check for association between hybridity and CWD status within the disease‐matching group. Analyses were done in R using the *glm* and *fisher.test* functions from the *stats* package (R core team, 2020). Likelihood‐ratio test and Rao's score test were calculated using the package *LogisticDx* (Dardis, 2015) (Table 2).

**TABLE 2:** Summary and significance testing of a logistic regression model predicting hybrid status from species, sex and CWD infection status

Parameter	*β* estimate	SE	Wald (Z value)	*p* (Wald Z)	*p* (LRT)	*p* (Rao score)	Odds ratio
Intercept	−5.725	0.377	−15.194	NA	NA	NA	NA
Species (WT = 1, MD = 0)	1.463	0.418	3.500	<0.001	<0.001	<0.001	4.318
Sex (male = 1, female = 0)	−0.296	0.420	−0.705	0.481	0.478	0.157	0.744
CWD status (pos = 1, neg = 0)	0.793	0.560	1.417	0.157	0.192	0.004	2.210

Note: All variables were binary. Species was re‐coded using the results from our own STRUCTURE analysis. Hybrid status, the response variable, is influenced by species with white‐tailed deer being over‐represented. Neither sex nor CWD infection status held any association with hybrid status. The same result was found when CWD status was compared within the disease‐matching group via a Fisher's test.

## 3 RESULTS

### 3.1 Genotyping

Three samples from the disease‐matching group failed genotyping, leaving 987 hunter‐submitted samples with call rates averaging 99.82%. Sampling groups break down as follows: 404 demographic‐matching, 497 disease‐matching (248 CWD‐ and 249 CWD+), 70 foothills, 16 ambiguous species, and 72 *a priori* hybrids with known pedigrees, plus 4,996 individuals previously genotyped at 10 microsatellite loci. See Figure 1 for sampling locations and Table 1 for a summary of sample group membership.

### 3.2 Simulated admixture analysis

**FIGURE 3:**
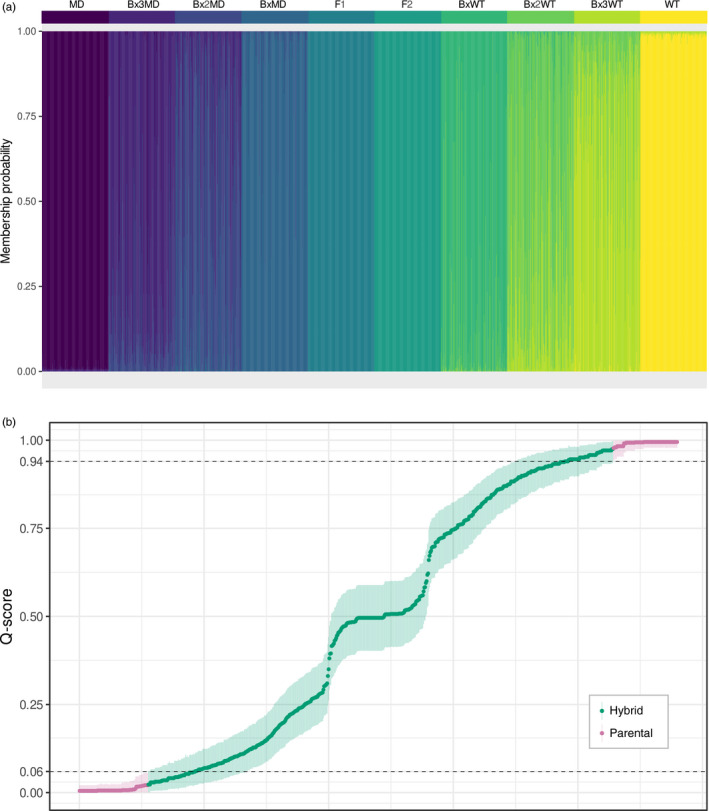
Hybrid assignments of simulated populations at two different introgression levels by two different programmes. (a) *NewHybrids* assignment of a population with 10 genotype frequency classes, including three backcross generations. Each vertical bar represents one individual's probability of belonging to a particular category, denoted by colour. (b) *STRUCTURE* analysis of a population with 12 genotype frequency classes, including a fourth backcross. Q‐score thresholds were set at 0.06 and 0.94; individuals were identified as hybrids if the 90% credibility interval of their Q‐score overlapped this region. See Figure 2 for derivation of these thresholds and their equivalents in the microsatellite suite

Simulated hybrid populations were used to determine the efficacy of detection of various levels of introgression by different computational methods when paired with our specific data sets (Figure 3). Here, two statistics defined in Vähä and Primmer (2006) are useful to evaluate hybrid assignment: ‘efficiency’ refers to the proportion of individuals correctly assigned (correct assignments / total individuals belonging to a particular class), and ‘accuracy’ refers to the proportion of assignments that are correct (correct assignments / total assignments to a particular class). Note that the efficiency statistic also captures the few backcross individuals with parental genotypes (as described in Boecklen and Howard 1997).

Hybrid identification was most successful for individuals genotyped at 40 species‐discriminating SNP loci. In a population with 10 genotype frequency classes (including three backcross generations), *NewHybrids* achieved a hybrid accuracy of 1.0 with zero false positives (parental species mistaken as hybrids). When the 8 hybrid classes were pooled, *NewHybrids* correctly assigned 1,578 of 1,600 hybrids for an efficiency of 0.986. With the genotype frequency classes kept separate, the class with the lowest accuracy was also the deepest level of introgression: third backcross MD. The lowest efficiency class was second backcross MD (Figure 3a). In a population with 12 genotype frequency classes, including a fourth backcross, Q‐score thresholds of 0.06 and 0.94 produced a hybrid accuracy of 1.0 (zero false positives) and efficiency of 0.932 (Figure 3b). Widening the thresholds introduced false positives and narrowing them decreased efficiency (Figure 2). Note that this method does not recognize individual generations but describes a general distinction between hybrid and parental.

Resolution was reduced in the microsatellite data set. In the population with 6 genotype frequency classes (i.e. one backcross generation), *NewHybrids* achieved a combined hybrid accuracy of ~1.0 (1 of 400 parental species was assigned as a hybrid) and efficiency of 0.99. In the population with 8 genotype frequency classes (including two backcross generations), Q‐score thresholds at 0.26 and 0.74 identified hybrids with an accuracy of 1.0 and efficiency of 0.885 (Figure 2).

### 3.3 Empirical admixture analysis

Of the 987 samples genotyped at diagnostic SNPs, 3 were identified as hybrids by *NewHybrids* for a rate of 0.30%. They included a first backcross male MD from the demographic‐matching group, a third backcross male MD that tested positive for CWD, and a third backcross female WT from the ambiguous species group. These 3 individuals, plus 12 more, had Q‐score CIs between 0.06 and 0.94 (1.5% of the total). These 12 additional deer included 6 from the demographic‐matching group (2 of which were from the same cell: 1 MD‐like and 1 WT‐like), 2 female WT from the foothills of the Rockies, 2 whose species was ambiguous (1 female and 1 that was left unsexed) and a female MD that tested positive for CWD. Mean (±SE) Q‐score CI width was 0.00553 ±0.000436. The deepest level of introgression of the *a priori* hybrids was the third backcross generation; all were assigned correctly as hybrids by both *NewHybrids* and *STRUCTURE*, and the specific hybrid category identified by *NewHybrids* was within one generation of the known pedigree for 96% of individuals (69/72; Table S3).

In 4,996 individuals genotyped at microsatellite loci, 3 were identified as hybrids by *NewHybrids* for a rate of 0.06%. Two were F2 s, and one was a backcross MD. 31 more had Q‐score CIs between 0.26 and 0.74 (0.68% of the total). See Table S1 for microsatellite summary statistics. Mean Q‐score CI width was 0.0173 ±0.00055. Geographically, hybrids from both data sets appear to be randomly distributed (Figure 1).

### 3.4 Modelling introgression direction

The logistic regression model comparing hybrid status to species, sex, and CWD status found only the species term to be significantly predictive according to Wald, likelihood ratio, and score tests (Table 2). Hybrid deer were disproportionately WT‐like (*p *< 0.001, OR: 4.32); their odds of occurring were 4.32× that of MD‐like hybrids. Fisher's exact test indicated no association between CWD status and hybrid status within the disease‐matching sample group (*p* = 0.96).

## 4 DISCUSSION

Rates of hybridization were determined for a sympatric population of mule and white‐tailed deer. Here, we analysed a hybrid zone with conservative and relaxed introgression estimators in tandem to capture low and high ends of a range of hybridization rates, respectively. The 987 deer genotyped at species‐discriminating SNPs included 3–15 hybrid individuals, and the 4,996 deer genotyped at microsatellite loci contained 3–34 hybrids. While interspecific admixture continues to persist in western Canada, it appears to be heavily suppressed and unlikely to exceed the high‐end estimates provided here. The highest rate of hybridization came from the STRUCTURE admixture analysis of those individuals with SNP genotypes, that is the samples submitted by hunters as part of a provincial CWD monitoring programme. Hybridization appears slightly more common in the SNP‐assayed data set because those loci offer increased power over the microsatellite markers to detect lower levels of introgression but estimates were still modest at 0.3 to 1.5%. The microsatellite suite supported the results of the SNPs in a robust sample size; its reduced sensitivity to interspecific admixture was reflected in the proportionately lower hybridization rates.

We also explored the direction of introgression and conditions that may promote or deter hybridization. WT was significantly over‐represented in our hybrid sample, meaning hybrids were disproportionately WT‐like. This suggests a slow introgression of MD alleles into the WT population. The cause of this trend has a number of possible explanations and could be a direction for future study. Given the rarity of hybridization events in the wild and the reduced fecundity of males (Wishart et al., 1988), matings between two hybrid deer seem unlikely. Rather, the directional asymmetry may be the result of a proclivity on the part of the hybrid offspring for choosing WT mates. For example, recall that deer social structure is matrilineal in nature; the foundational social unit is the doe–fawn relationship (Hawkins & Klimstra, 1970; Kie et al., 2002). Perhaps F1 offspring tend to seek out familiar mates conspecific to their own social group (and that of their does), in which case the relative prevalence of WT‐like hybrids is an indication that the doe in a heterospecific mating pair tends to be WT (i.e. WT doe x MD buck, as in Bradley et al., 2003; Carr et al., 1986; Hughes & Carr, 1993; Wishart et al., 1988). Alternatively, the recent pattern of widespread WT encroachment into habitats historically occupied by MD may cause a demographic swamping of MD alleles (as described in West Texas by Ballinger et al., 1992; Wiggers & Beasom, 1986). Of course, these theories are not mutually exclusive and may co‐occur with other factors.

No F1s were identified in this study. All hybrids from wild populations were post‐F1 backcrosses and, moreover, tended to be backcrossed multiple times with Q‐scores approaching 0 or 1. This tendency towards deeper introgression is consistent with previous findings that early‐generation hybrids appear maladapted compared with their parental forms. Lingle (1993) found evidence that early‐generation hybrids may suffer increased predation because of biomechanically inefficient escape gaits. Wishart et al. (1988) concluded that MD × WT hybrids observe Haldane's rule, which predicts that heterozygote breakdown will affect the heterogametic sex more adversely than the homogametic sex (Haldane, 1922). But the same study also observed increased maturation and quality of spermatozoa in later‐generation hybrids compared with F1s, indicating that male fertility returns more with each backcross generation. The result is a bimodal distribution of hybridity where the majority of individuals are effectively parental species and only a small minority can be called interspecific hybrids. While acknowledging a small hybrid sample size, we submit that the deficiency of F1s observed here supports the established consensus that early‐generation hybrids suffer from heterozygote breakdown and extend this principle to wild populations. If true, the system may be said to be a tension zone where hybrid populations are maintained by a balance of migration and selection against hybrids (Hewitt, 1988; Hu, 2005).

Similar low rates have been reported previously: 1–2% in south‐western United States (Derr, 1991), 2% in Montana (Cronin et al., 1988), and 3–4% in Alberta (Hughes & Carr, 1993). Rates as high as 6% have been observed in Alberta (Hornbeck & Mahoney, 2000) but in that instance all hybrids were found in one location where human‐mediated environmental disturbance may have fostered increased hybridization (Todesco et al., 2016). Although we found no such hotspots in this study, similar patterns have been confirmed in the comparable sika (*Cervus nippon*) × red deer (*C*. *elaphus*) system in the UK where both species experience little predation pressure (Senn & Pemberton, 2009; Senn et al., 2010). Localized hotspots are characteristic of mosaic hybrid zones: areas of species overlap with a patchy distribution of some ecological factor that favours one species over the other (Bierne et al., 2002, 2013; M'Gonigle & FitzJohn, 2010; Ross & Harrison, 2002).

Only two hybrids tested positive for CWD. This does not exceed expectations based on chance given the overall hybridization rates and number of CWD+ individuals sampled (Fisher's exact test *p *> 0.05). Hybridization events may, in theory, spread CWD when they do occur but because hybrids are so infrequent they likely play a reduced role, if any. We should, however, acknowledge two caveats: (a) that heterospecific mating appears to have a low success rate (Wishart et al., 1988), suggesting that total mating attempts will outnumber F1 hybrid individuals, and (b) that CWD can transmit horizontally and vertically (Miller et al., 2000; Williams, 2005). Future work on the propensity of heterospecifics to attempt mating—regardless of reproductive success—may have implications for horizontal, interspecific CWD transmission (Cullingham et al., 2010, 2011; Nalls et al., 2013).

Although wild deer populations of Alberta have low hybridization rates, other populations may vary. Captive populations such as cervid farms are unlikely to stock both species in the same enclosure, but farmed animals can sometimes contribute to local allele frequencies and gene flow via escapes from the facility (Russo et al., 2019). Populations in other parts of the hybrid zone may vary as well; MD and WT are sympatric throughout much of western North America. The ecological nuances of different habitats can cause hybrid zones to produce a variety of outcomes, even between multiple instances of the same parental species (Gompert & Buerkle, 2016). For wildlife managers and researchers interested in areas of this hybrid zone outside of Alberta, the resources and methods reported here will be applicable and may draw different results.

We have shown that MD and WT of western Canada hybridize at a similar, if slightly lower rate than that reported in other parts of the hybrid zone and that the few hybrids found were disproportionately WT‐like (Cronin et al., 1988; Derr, 1991; Hughes & Carr, 1993; Senn & Pemberton, 2009; Senn et al., 2010). Interspecific reproductive barriers of these species are bidirectionally semipermeable but hybrids are still a rare occurrence. When a hybridization event does take place the resulting offspring is likely the target of more intense selective pressure than that applied to its parents or backcrossed descendants. Phylogenetic lineages in Alberta appear to remain mostly intact and well‐structured.

## CONFLICT OF INTEREST

None declared.

## Supporting information

Table S1

Table S2

Table S3

## Data Availability

All data discussed in this manuscript, including SNP genotypes and R script for simulating hybrids and evaluating assignment efficacy, are publicly available on Dataverse (https://doi.org/10.7939/DVN/FSRWR4).
